# Analysis of mRNA abundance for histone variants, histone- and DNA-modifiers in bovine *in vivo* and *in vitro* oocytes and embryos

**DOI:** 10.1038/s41598-018-38083-4

**Published:** 2019-02-04

**Authors:** J. Duan, L. Zhu, H. Dong, X. Zheng, Z. Jiang, J. Chen, X. C. Tian

**Affiliations:** 1 Department of Animal Science, University of Connecticut, Storrs, CT 06269 USA; 2 Xinjiang Academy of Animal Science, Urumqi, Xinjiang P. R. China; 3 Present Address: School of Animal Sciences, Louisiana State University, Baton Rouge, LA USA

## Abstract

Transcript abundance of histone variants, modifiers of histone and DNA in bovine *in vivo* oocytes and embryos were measured as mean transcripts per million (TPM). Six of 14 annotated histone variants, 8 of 52 histone methyl-transferases, 5 of 29 histone de-methylases, 5 of 20 acetyl-transferases, 5 of 19 de-acetylases, 1 of 4 DNA methyl-transferases and 0 of 3 DNA de-methylases were abundant (TPM >50) in at least one stage studied. Overall, oocytes and embryos contained more varieties of mRNAs for histone modification than for DNA. Three expression patterns were identified for histone modifiers: (1) transcription before embryonic genome activation (EGA) and down-regulated thereafter such as *PRMT1*; (2) low in oocytes but transiently increased for EGA such as *EZH2*; (3) high in oocytes but decreased by EGA such as *SETD3*. These expression patterns were altered by *in vitro* culture. Additionally, the presence of mRNAs for the TET enzymes throughout pre-implantation development suggests persistent de-methylation. Together, although DNA methylation changes are well-recognized, the first and second orders of significance in epigenetic changes by *in vivo* embryos may be histone variant replacements and modifications of histones.

## Introduction

The basic unit of chromatin, the nucleosome, consists of the histone octamer wrapped by 147 bp of DNA. In eukaryotes, the histone octamer is comprised of two of each core histone proteins (H2A, H2B, H3 and H4). Nucleosomes are further packaged and stabilized by linker DNA and linker histone proteins (H1/H5)^1^.

During cell division, the continuous demand for histone proteins to package newly replicated DNA is satisfied by newly synthesized histones and those recycled from the parental cells^2^. The production of histones is either replication-dependent or independent. Canonical histones are expressed only during the S phase of the cell cycle and their incorporation into chromatin is tightly coordinated with DNA replication^2^. Instead of the poly(A) tails providing stability, their transcripts contain a 3′ end stem-loop structure which contributes to their stability^3,4^. Replication-independent histones, however, are transcribed throughout the cell cycle and their incorporation into nucleosomes is independent of DNA synthesis^5^. These histones are also known as replacement histone variants and are translated from polyadenylated mRNAs. These mRNAs may originally contain introns before fully maturing, and in some cases, undergo alternative splicing which allows them to encode distinct isoforms^6,7^. In mammals, most histones have multiple variants. However, to date, no replacement variants have been identified for H4^8^. H2B is yet another histone variant that is understudied, and no bovine histone H2B variant has been annotated so far.

In mice, sperm replace histones with protamine in order for the DNA to be densely compacted. After fertilization, the male pronucleus undergoes protamine-to-histone exchange through the incorporation of maternal histone variants, mainly H3.3, an H3 variant^9^. The resulting pre-implantation embryos undergo even further and more dynamic changes in chromatin composition as a result of replacement by other histone variants^10,11^. These observations were consistent with findings from mouse somatic cell nuclear transfer (SCNT) studies, which reported rapid replacement of H2A and H3 histones of the donor cells by oocyte-stored H2AFX and H3 variants, respectively^12^. Together, these mouse studies demonstrated that the incorporation of oocyte-stored histone variants into the genome of donor somatic cells or sperm is essential for modulating chromatin structures and gene expression of the newly formed embryos.

Embryonic genome activation (EGA) is the process during which the embryonic genome is actively transcribed^13,14^. Both the timing of EGA and proper activation of specific genes are essential to embryonic development. The timing of EGA is very different among mammalian species and is usually correlated to the speed of embryonic development^14^. For example, EGA occurs at the 2-cell stage in mice^15^ and between 4–8 cell stage in humans^16^. Although EGA in bovine *in vitro* embryos occurs between the 8–16 cell stage^17^, *in vivo* embryos actively transcribe their genome between the 4–8 cell stage^18,19^.

Gene expression changes are also profoundly regulated by post-translational modifications of histones, such as acetylation, methylation, phosphorylation, ubiquitylation, sumoylation and ADP-ribosylation^20^. Among these, acetylation and methylation of lysine residues by acetyl-transferases and methyl-transferases, respectively, are the most studied modifications. These modifications could also be reversed by de-acetylases and de-methylases^21,22^. DNA methylation is another mechanism of transcription regulation. It has recently been reported that maternal stored nuclear reprogramming factors, such as DNA de-methylation enzymes by oxidation, ten-eleven translocation 3 (*TET3*), along with histone variants and histone post-translational modification enzymes, contribute to embryonic genome activation and embryonic totipotency^23^.

The roles of histone variants and histone/DNA modifications during pre-implantation development has been a major field of research^5,8,24^. Previous studies employing semi-quantitative real time-PCR revealed altered expression patterns of histone and DNA modifying enzymes in bovine oocytes and embryos from assisted reproductive technologies, such as *in vitro* fertilization and SCNT^25,26^. However, a comprehensive profile of these important epigenetic regulators from bovine *in vivo* embryos has not been published. Here, we report data-mining results from an RNA-Seq study of bovine *in vivo* produced oocytes and pre-implantation embryos^18^. We document distinct waves of changes in mRNAs for histone variants as well as histone/DNA modifying enzymes. Although there have been widely recognized DNA methylation dynamics in early embryos, we found much higher levels as well as more varieties of mRNAs for histone variants and for the modification of histones than for DNA, suggesting a bigger/more important roles of histone modifications in pre-implantation embryonic development. Moreover, we also compared the differences of these transcripts between *in vivo* and *in vitro* produced embryos and identified alterations of genes expression by *in vitro* culture.

## Results

### Registry of mRNAs for Histone Variants, and Histone/DNA Modifying Enzymes

A summary of mRNAs for replication-independent histone variants, histone/DNA modifying enzymes that have been annotated in the bovine as well as those that were detected in bovine oocytes and pre-implantation embryos is shown in Table 1. The mRNAs for a total of 116 out of the 141 annotated genes were detected (mean TPM >1). Epigenetic regulations in early development via histone variants (10 variants) and histone modification (100 enzymes) were more diverse than via DNA modifications (6 enzymes; Table 1). Among the 116 detected transcripts, the mRNAs for 30 different epigenetic modifiers (or 25.9%, 30/116) were abundant (Mean TPM >50) in at least one of the stages examined. Although more histone methylation modifiers were detected (64 out of 116), only 20.3% (13/64) were abundantly expressed. On the other hand, higher detection percentage of histone acetylation modifiers (10/36 or 27.8%) were abundant. It is also worth-noting that one-third of mRNAs detection for histone variants were at extremely high levels (TPM >1,000), while none of the histone/DNA modifiers reached such levels. Together, these observations showed that in pre-implantation development, epigenetic changes may occur mainly through histone variant replacements, secondly, histone modifications, and lastly, DNA modifications.

**Table 1:** Registry of mRNAs for histone variants, and histone/DNA modifying enzymes during bovine early embryogenesis.

mRNA	# Annotated in bovine	# (%) Detected (TPM > 1)	# (%) Abundant (TPM > 50)	# (%) Highly abundant (TPM > 1,000)
Histone variants	14	10 (71.4)	6 (60.0)	3 (30.0)
Histone modifiers				
Histone methyl-transferases	52	40 (76.9)	8 (20.0)	0
Histone de-methylases	29	24 (82.8)	5 (20.8)	0
Histone acetyl-transferases	20	19 (95.0)	5 (26.3)	0
Histone de-acetylases	19	17 (89.5)	5 (29.4)	0
DNA modifiers				
DNA methyl-transferases	4	3 (75)	1 (33.3)	0
DNA de-methylation by oxidation	3	3 (100)	0	0
Total	141	116 (82.3)	30 (25.9)	3 (2.6)

### Abundance of mRNAs for histone variants

**Figure 1:**
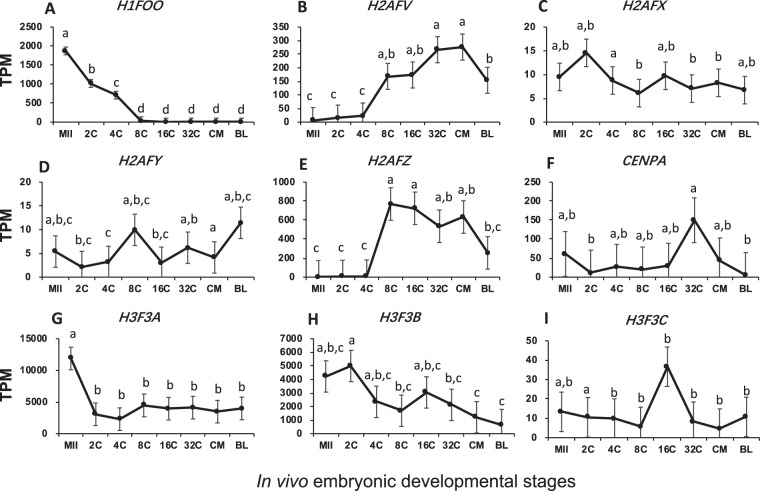
Transcript abundance of bovine histone variants during *in vivo* preimplantation development (Mean ± SEM). MII = mature oocytes; 2C to 32C = 2- to 32-cell embryos; CM = compact morula; BL = blastocyst. Different letters above error bars indicate significant pair-wise differences.

Among those annotated, the transcripts for only one bovine linker histone variants, *H1FOO*, were detected. At a TPM value of 1,872.3 (Fig. 1A), this oocyte-specific linker H1 was among the most abundant mRNAs in oocytes. *H1FOO* transcripts dropped dramatically after fertilization and after the 8-cell stage were barely detectable, presumably due to RNA degradation and lack of new transcription. Together, these data showed that it took as few as 3 rounds of cell divisions, or 2 days, for complete degradation of a large amount of transcripts in early embryos. Coincidentally, this is also the timing for bovine EGA^18,19^ in embryos developed *in vivo*. It is possible that H1FOO depletion facilitates the opening of the embryonic chromatin. In contrast, there was no detection of mRNA for other linker histone variants, such as *H1F0*, the testis-specific *H1FNT* or the somatic-specific *H1FX*, in all samples/stages.

Six H2A variants have been annotated in the bovine, the mRNA for five of them were detected: *H2AFJ* was barely present (Table S1), *H2AFX/Y* was maintained at low yet relatively constant levels across stages (Fig. 1C,D; P = 0.243, 0.167, respectively). In contrast, *H2AFV/Z* was abundant in at least one stage (Fig. 1B,E) and was either actively transcribed as a result of EGA between the 4- and 8-cell stages in bovine *in vivo* embryos. It was noteworthy that the mRNAs for *H2AFV* and *H2AFZ* were the most abundant among all detected H2 variants. *H2AFZ*, with a TPM value as high as 769.3 at the 8-cell stage (Table S1, Fig. 1E), averaged around 100X the level of the other H2 variants with the exception of *H2AFV*, suggesting its importance in post-EGA development.

For histone H3, there is the detection of four annotated variants, with high expression in three of them. Among the three H3 variants with high TPM values (i.e., *H3F3A*, *H3F3B* and *CENPA*), *H3F3A* was stored at extremely high levels in oocytes (~12,000 TPM, Fig. 1G) but decreases more than 3.8 times after fertilization. During embryonic development its levels remained high at around 5,000 TPM. *H3F3B* had an overall downward change across stages even though the oocytes contained large quantity of this transcript (Fig. 1H). Relative steady expression of the other abundant H3 variant, *CENPA*, along with *H3F3C*, was seen during early cleavage stages (p = 0.385, 0.180 respectively). They did, however, undergone a burst of expression at the 32- and 16- cell stages, respectively (Fig. 1F,I).

### Levels of transcripts for histone modifying enzymes

Based on the current bovine genome assembly and annotation, we generated a list of genes that post-translationally modify histones (Table S2–5). The mRNAs for the majority of these genes were detected (100/120) in our bovine RNA-Seq data (Table 1).

Among histone methyl-transferases, three main patterns of changes were observed:

1. Transcription before EGA and down-regulated thereafter. These included *PRMT1*, which mono- and di-methylates Arg-4 of histone H4, *SUV420H1*, which trimethylates Lys-20 of histone H4, and *LOC534913*, also known as mariner-like transposase which is predicted to be a histone lysine methyltransferase. Their mRNAs were high before EGA, likely from storage in the oocytes and active transcription occurred at the 2- and 4-cell stages. Their transcription was turned off subsequently after EGA (Fig. 2A–C). Interestingly, although similar changes were seen for *PRMT1* and *SUV420H1*, they methylate different residues of histone H4 and exert different effects on gene expression. *PRMT1* leads to transcription activation while *SUV420H1* leads to transcription repression (http://www.uniprot.org).

**Figure 2:**
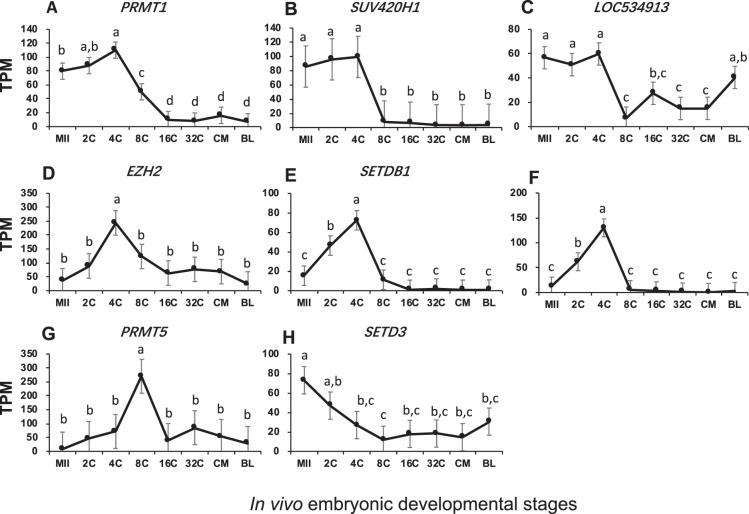
Transcript abundance of bovine histone methyltransferases during *in vivo* preimplantation development (Mean ± SEM). MII = mature oocytes; 2C to 32C = 2- to 32-cell embryos; CM = compact morula; BL = blastocyst. Different letters above error bars indicate significant pair-wise differences.
2. Low in oocytes but increased transiently for EGA. These included *EZH2*, which methylates Lys-9 and Lys-27 of histone H3, *SETDB1/SUV39H2*, which specifically trimethylates Lys-9 of histone H3, and *PRMT5*, which methylates Arg-8 of histone H3. They were transiently transcribed around EGA, reaching peak TPM values at the 4- or 8-cell stage (Fig. 2D–G). It has been reported that the histone methylation activity by these four enzymes represents a specific tag for transcription repression (http://www.uniprot.org).
3. High in oocytes but decreased from EGA. *SETD3*, which methylates Lys-4 and Lys-36 of histone H3 and acts as a transcriptional activator (http://www.uniprot.org), was inherited in oocytes and degraded dramatically after fertilization likely because of no active transcription (Fig. 2H).

**Figure 3:**
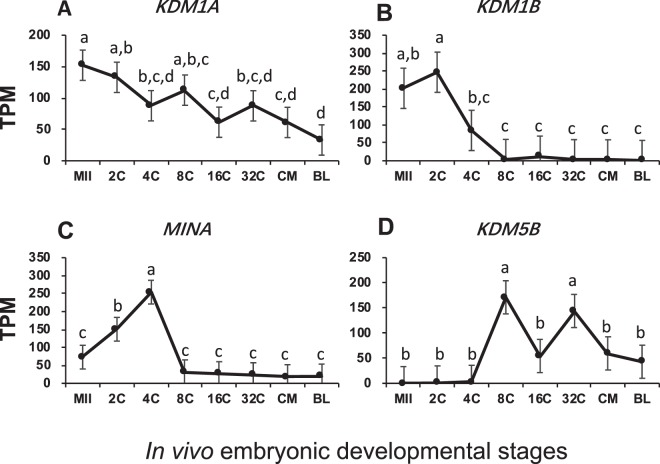
Transcript abundance of bovine histone demethylases during *in vivo* preimplantation development (Mean ± SEM). MII = mature oocytes; 2C to 32C = 2- to 32-cell embryos; CM = compact morula; BL = blastocyst. Different letters above error bars indicate significant pair-wise differences.

Like histone methyltransferases, oocytes abundantly inherited histone demethylases including *KDM1A/B* (Fig. 3A,B). *KDM1A* can induce both transcription activation and repression by de-methylating Lys-4/9 of H3. Its mRNA degraded gradually after fertilization and this pattern of changes suggested that the early embryos may actively maintain de-methylation of H3 (Fig. 3A). On the other hand, *KDM1B*, which also de-methylates Lys-4 of histone H3, actively maintained its mRNA at the 2-cell stage but decreased dramatically before EGA (Fig. 3B). Active transcription of two other abundant histone demethylases, *MINA* and *KDM5B*, occurred before and after EGA, respectively (Fig. 3C,D). Together, these data suggested that except for *KDM5B*, de-methylation of histones mainly occurred on H3 before EGA.

In summary, the mRNA levels for histone methylation enzymes underwent dramatic changes mainly before and around EGA. Overall there as more mRNAs for histone methylation than for de-methylation.

**Figure 4:**
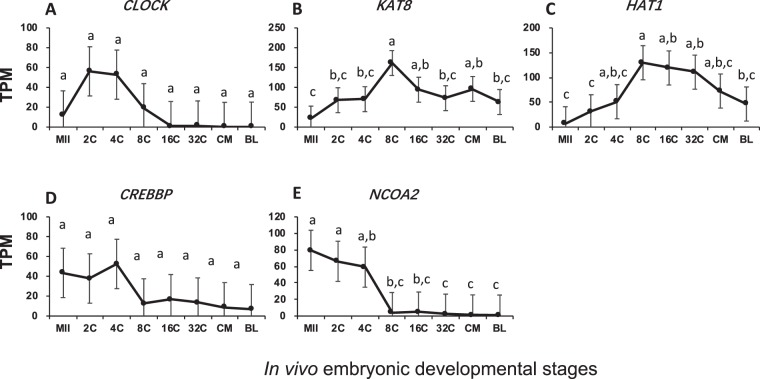
Transcript abundance of bovine histone acetyltransferase during *in vivo* preimplantation development (Mean ± SEM). MII = mature oocytes; 2C to 32C = 2- to 32-cell embryos; CM = compact morula; BL = blastocyst. Different letters above error bars indicate significant pair-wise differences.

**Figure 5:**
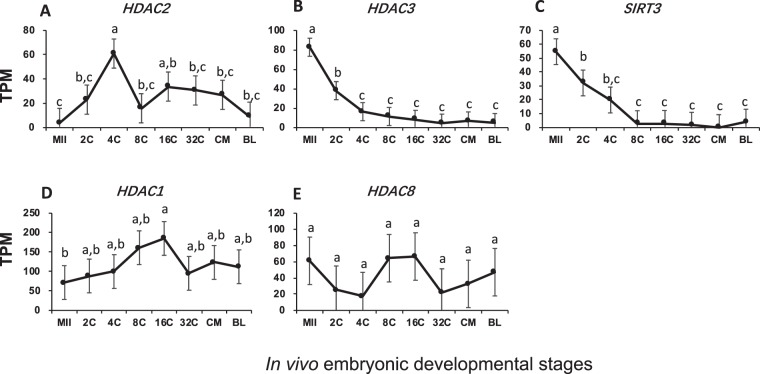
Transcript abundance of bovine histone deacetylases during *in vivo* preimplantation development (Mean ± SEM). MII = mature oocytes; 2C to 32C = 2- to 32-cell embryos; CM = compact morula; BL = blastocyst. Different letters above error bar indicates pair-wise differences.

For histone acetylation, abundant mRNAs for five of histone acetyltransferases and deacetylases were found (Figs 4 and 5). Maintenance of modest levels of mRNAs for histone acetyltransferases, *CLOCK*, *KAT8*, *HAT1* and *CREBBP*, was seen across all stages, despite minor and insignificant changes (Fig. 4A–D). In contrast, transcripts of NCOA2, which binds with CREBBP to actively acetylate lysine residues on core histone tails, were inherited from oocytes at a moderate level with depletion occurring from EGA (Fig. 4E).

Three different expression patterns were also seen in histone deacetylases: (1) active transcription of *HDAC2* occurred before EGA (Fig. 5A); (2) abundant inheritance of *HDAC3* and *SIRT3* transcripts from oocytes with active depletion through early development (Fig. 5B,C); and (3) relatively constant and moderate levels of transcripts for *HDAC1/8* across stages (Fig. 5D,E). Unlike histone methylation, which can lead to either gene expression or repression, histone acetylation/deacetylation always open/close chromatin structure and activate/repress gene expression. The drop in the mRNAs for deacetylases likely prepared the embryos for more open chromatin at later stages. Also of note, mRNAs for histone acetylation/deacetylation were always present at modest levels across preimplantation development (Figs 4 and 5), while those for histone methylation/demethylation were mainly present before/around EGA (Figs 2 and 3).

### Abundance of mRNAs for DNA modifying enzymes

Global embryonic DNA methylation has been reported to undergo dramatic changes first through active and then passive de-methylation, finishing with *de novo* active methylation^27–29^. The dynamics of mRNAs for DNA modifiers found here corresponded to the reported changes and also provided unique features for bovine *in vivo* embryos.

**Figure 6:**
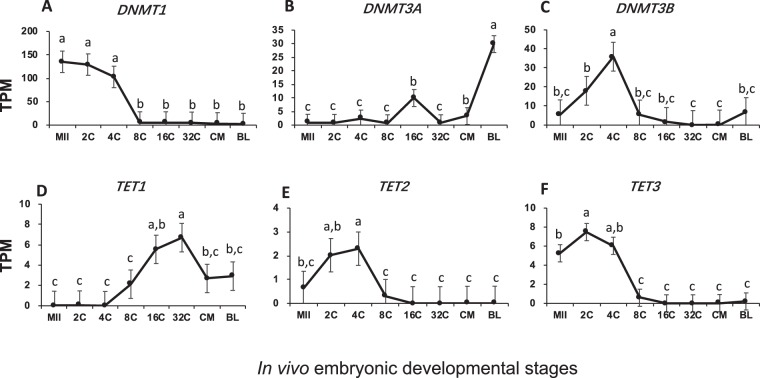
Transcript abundance of bovine DNA modifiers during *in vivo* preimplantation development (Mean ± SEM). MII = mature oocytes; 2C to 32C = 2- to 32-cell embryos; CM = compact morula; BL = blastocyst. Different letters above error bars indicate significant pair-wise differences.

Transcripts for *DNMT1* (Fig. 6A), a DNA methylation maintenance enzyme, were the most abundant among all DNA modifiers. The high storage of mRNA for *DNMT1* in oocytes progressively depleted after fertilization. After EGA very low levels of *DNMT1* mRNA remained, allowing embryos to passively de-methylate through cell divisions. In contrast, the mRNA for *DNMT3A* (Fig. 6B), which is responsible for DNA *de novo* methylation, was almost undetectable before the 16-cell stage but became the predominant DNA modifying mRNA at the blastocyst stage. These observations suggest that the first stage of differentiation in the blastocyst requires new methylation of DNA. *DNMT3B* transiently increased its mRNA levels during EGA which subsequently declined to basal levels (Fig. 6C), suggesting the role of new methylation for EGA. The protein DNMT3L is catalytically inactive but is required for establishment of proper genomic imprinting by possibly facilitating *de novo* DNA methylation by *DNMT3A* and *DNMT3B*^30^. Its mRNA, however, was not detected in bovine *in vivo* oocytes and embryos (Table S6).

The TET family is so far the best characterized enzymes that actively initiate the removal of the methyl group from 5′ methylated cytidine (5 mC) to 5-hydroxymethylcytosine (5 hmC), 5-formylcytosine (5 fC) and 5-carboxylcytosine (5 caC) through oxidation. These modified cytidines are later replaced with unmodified cytosine via the thymine DNA glycosylase- (TDG) base excision repair pathway^31,32^. The mRNAs for two of the three TET genes, *TET1* and *TET3*, were found to have a relatively significant presence in bovine post-EGA embryos and oocytes/pre-EGA embryos, respectively (Fig. 6D,F). Another TET member, *TET2*, was barely detected during pre-implantation development (Fig. 6E).

**Figure 7:**
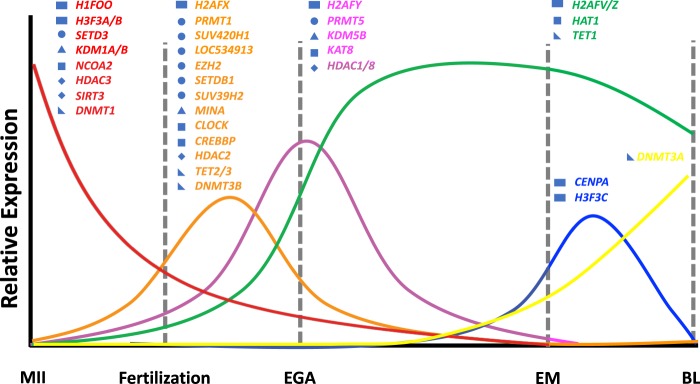
Patterns of changes in mRNAs of major histone variants and modifiers for histone and DNA at landmark stages of embryo development. The Y-axis does not represent the absolute levels of each transcript. MII = mature oocytes; EGA = embryonic genome activation; EM = early morula; BL = blastocyst. Histone variants: ; Histone methyltransferases: ; Histone demethylases ; Histone acetyltransferase ; Histone deacetylases: ; DNA modifiers: .

In total, six patterns of gene expression dynamics in histone variants, histone and DNA modifiers were seen in our study (Fig. 7). These were: (1) peaked in oocytes but decreased by EGA (red line), (2) transcription before EGA and down-regulated thereafter (orange line), (3) peaked at EGA (pink line), (4) activated post-EGA (green line), (5) peaked around the morula stage (blue line), and (6) peaked at the blastocyst stage (yellow line).

### Gene expression patterns of epigenetic modifiers in *in vitro* produced oocytes and embryos

**Figure 8:**
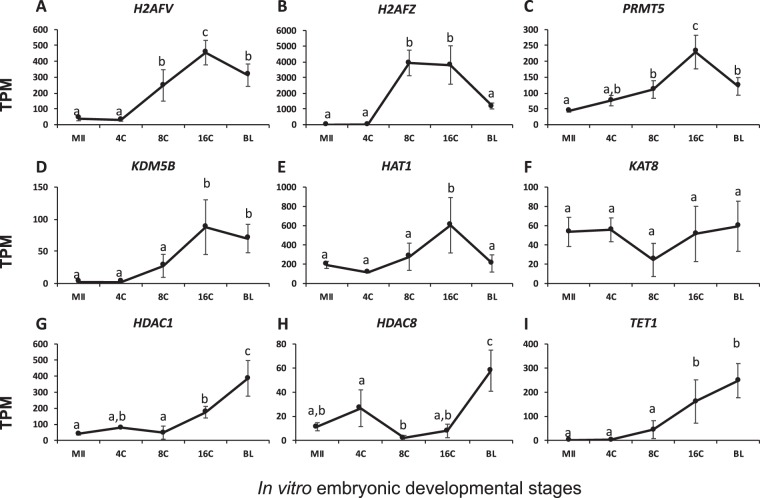
Transcript abundance of bovine histone and DNA modifier during *in vitro* preimplantation development (Mean ± SEM). MII = mature oocytes; 4C to 16C = 4- to 16-cell embryos; BL = blastocyst. Different letters above error bars indicate significant pair-wise differences.

Transcript abundance of bovine histone variants and histone/DNA modification proteins from *in vitro* produced oocytes and embryos is summarized in Table S7. The major difference between *in vitro* and *in vivo* produced bovine embryos was the timing of EGA, 8–16 cell stage In *in vitro* embryos^17^ and 4–8 cell stage in *in vivo* embryos^18^. Among the genes analyzed above, nine were different between the two groups. They were: *PRMT5*, *KDM5B*, *KAT8*, *HDAC1*, *HDAC8*, *H2AFV*, *H2AFZ*, *HAT1*, and *TET1*. Five of these, *H2AFV*, *H2AFZ*, *PRMT5*, *KDM5B*, and *HAT1*, had *a* delay in their peak expression from the 8-cell stage in *in vivo* embryos to the 16-cell stage in *in vitro* embryos (Fig. 8A–E). Moreover, the expression dynamics of the rest of the four genes, *KAT8*, *HDAC1*, *HDAC8*, and *TET1*, were different compared to those in the *in vivo* data (Fig. 8F–I).

### Validation of RNA-Seq data by qRT-PCR

All relative transcription levels were confirmed with the exception of one comparison, *KANSL2* 4-cell vs. blastocyst (Table 2). Only a difference of 2-fold change or Log2 (fold change) of greater than 1 was regarded as significantly changed. We found no significant changes in *KANSL2* levels using RNA-seq [Log2 (fold change) = 0.3)], yet qPCR found a significant increase [Log2 (fold change) = 1.6)].

**Table 2:** Log_2_ (fold change) of mRNA abundance of selected genes validated by qRT-PCR.

Gene ID	4-cell vs. 8-cell		4-cell vs. Blastocyst	
	RNA-Seq	qRT-PCR	RNA-Seq	qRT-PCR
*KANSL2*	1.56	2.9	−0.3	1.6
*ASH2L*	−4.2	−1.7	−2	−3.1
*H3F3B*	0.5	1.5	0	0.9
*HDAC1*	2.5	1.7	3.4	2.3
*SETDB1*	0.6	−0.5	0.2	0.2

## Discussion

Several lines of evidence support that histone H1 variant H1FOO is essential for *in vitro* meiotic maturation of mouse and bovine oocytes^33,34^. Over-expression of *H1FOO* in bovine oocytes prompted meiotic progression *in vitro*, while its down-regulation hindered oocyte maturation^33^. When injected into mouse germinal vesicle oocytes, antisense Morpholino oligonucleotides, which knocked down *H1FOO*, slowed the rate of *in vitro* maturation^34^. Replacement of somatic histone H1s by *H1FOO* was observed in embryos derived from both *in vitro* fertilization and SCNT^35,36^. Additionally, ectopically expressed *H1FOO* halted differentiation of embryonic stem cells into embryoid bodies^37^. These prior observations point to the importance of H1FOO in the maintenance of relatively quiescent chromatin structure before global gene activation. Our RNA-Seq data showed that the *in vivo* matured bovine oocytes stored high levels of mRNA for *H1FOO*, supporting the necessity for its high maintenance for maturation. Its subsequent and rapid disappearance in embryos suggest that H1FOO must be cleared from the early embryos before proper transition to EGA and differentiation could occur (Fig. 1A).

Incorporation of core histone variants are also essential during oocyte development and embryogenesis since the functions of these variants are more versatile than their linker counterparts for containing more post-translational modification sites^38,39^. For instance, using immunocytochemistry, Buhe *et al*. reported that H2A variants were deposited onto chromatin during mouse pre-implantation development^11^, and that H2AFX was the only abundant H2A variant after fertilization. While we also found major surges in mRNAs for H2A variants after fertilization, the specific variants were different than those reported in the mouse. In bovine pre-implantation embryos, *H2AFZ* and to a lesser extent *H2AFV* were pre-dominant after EGA (Figs 1B,E and 7). Yet, in the mouse, no incorporation of H2AFV or H2AFZ was observed until differentiation^40^. Thus, it appears that the dynamics of the H2A variants among mammalian species are not well-conserved. H2AFV and H2AFZ have been shown to prompt interactions among nucleosomes, which is critical in maintaining the high order structure of chromatin^41^, and essential for centromere assembly^42^. Although we observed an increase in mRNA for *H2AFX* at the 2-cell stage (Fig. 1C), which was likely a result of pre-EGA transcription, it was a minor H2A variant in the bovine as opposed in the mouse. H2AFX is reported to be largely phosphorylated during early embryogenesis in mice^43^, and its phosphorylation is required for the formation of the paternal pronucleus after fertilization in *Xenopus*^44^. Its role in bovine embryo development, however, may not be as critical as in the other species due to its lower mRNA levels. H2AFY, commonly known as macroH2A, has been shown to be actively replaced by other H2A variants from the maternal genome after fertilization^45^. It is also abundantly accumulated on the inactive X chromosome (XCI) in females and contributes to the long-term maintenance of the inactive state^46,47^. XCI in the bovine is completed during embryo elongation, a stage much later than in the mouse^44^. The levels of mRNAs for the gene *H2AFY* were present in early bovine embryos. It peaked in both the 8-cell and blastocyst stages. However, its overall expression levels were mostly lower than 15 TPM, suggesting that this transcription inhibitory H2A variant may be minimal during genome activation and blastulation (Figs 1D and 7).

In the mouse, the incorporation of H3F3A and H3F3B has been recognized as the marker for transcription activation and they preferentially integrate into the male pronuclei after fertilization^48^. This, at least in part, explains why paternal genome goes into transcription activation earlier^9^. These H3 variants maintain their occupation in the mouse embryos up to the blastocyst stage^23^. In the bovine embryos, however, both H3 variants *H3F3A/B* were extremely high in matured oocytes but then dramatically decreased, although their mRNA levels were still quite high compared to the other histone variants (Figs 1G,H and 7). These patterns of changes suggest that H3F3A/B were the predominant histone H3 variants in oocytes and subsequently their occupation was reduced and replaced by other H3 subtypes such as H3F3C and CENPA which temporarily peaked at the 16- and 32-cell stages (Figs 1F and 7). Such increases commensurate with the more centromere assembly and mitotic division activities at these stages^49,50^.

Canonical histone mRNAs have a unique stem loop-like 3′ end structure^4^, and were de-selected during poly(A) enrichment of RNA-seq library preparation. Thus, no replication-dependent histone transcripts should be detected here. However, we found that some canonical histone mRNAs were significantly abundant during bovine pre-implantation development (e.g. H2B, data not shown). The same observation was also reported in another RNA-Seq study in sheep, in which poly(A) selection was also employed^51^. By reviewing the mRNA sequences of these histones, we found that they all have high adenosine content after the 3′ stem loop structure, which would permit them to anneal with oligo-dT primers in library preparation and be detected.

In summary, our data suggest distinct waves of histone replacements by specific variants. It is likely that after fertilization and before EGA, the embryos first remove H1FOO and H3F3A/B, but add H2AFV/Z to the chromatin after EGA. To prepare for compaction, the embryos temporarily incorporate more H3F3C and CENPA into their nucleosomes.

The roles of histone post-translational modifications on chromatin remodeling and global gene expression have been well-documented. Yet, these roles are also very complex, especially for those by histone methylation. Mono-, di- or tri-methylation on different lysine residues of the histone tail leave chromatin structure in either open or closed state, resulting in transcription activation or repression^52^. Histone modifiers were the most diverse in bovine embryos and many had high transcript levels especially histone methyl transferases such as *EZH2*, *SUV420H1*, and *PRMT5* (Figs 2–5). Although each histone modifiers had their own distinct pattern of changes, their dynamic changes correlated with major events of embryo development such as EGA (*EZH2*, *SUV39H2*, *SUV420H1*, *LOC534913*, *MINA*, *HDAC3*, and *SIRT3*; Fig. 7), and pre-compaction (*PRMT5*, and *HDAC2*; Fig. 7). In mice, three expression patterns of chromatin modifiers were also found and classified as “maternal”, “ubiquitous”, and “zygotic”^49^, corresponding in our study to “inherited from oocytes and decreased after fertilization”; “relatively constantly expressed”; and “actively transcribed as a result of EGA”, respectively. In bovine early embryos, however, we noticed another main pattern “transcribed before EGA and decrease thereafter” (e.g. Figs 2A–C and 7) as a result of minor EGA^18^. The presence of this unique pattern is likely because of the longer duration of embryogenesis in the bovine than in the mouse.

Specific histone modifiers are linked to embryonic chromatin remolding and gene expression. *De novo* synthesis of EZH2 protein after fertilization was found to be essential to mouse embryo development because inhibition of EZH2 synthesis at the pronuclear stage caused retarded embryo growth and reduced blastocyst rates^53^. In bovine, a burst of transcription for the *EZH2* gene was observed at the 4-cell stage (Fig. 2D), which corresponded with the initiation of EGA in *in vivo* embryos^18^. Combined with fact that EZH2 trimethylates Lys-27 of histone H3 and leads to gene silencing, it would be of great interest to identify the specific genes that are turned off by EZH2, as well as to learn how the absence of these downstream genes initiates EGA and promotes early embryo development. Lys-9 of histone H3 is another potential methylation site. Trimethylation at this location serves as the loading dock for the heterochromatin organization protein HP1 and therefore is associated with transcription repression of the heterochromatin regions^54^. SETDB1 and SUV39H2 specifically trimethylates Lys-9 of histone H3. In bovine early embryos, we found a burst of expression for both of these histone methyltransferases around the 4-cell stage (Fig. 2E,F). These suggest that the formation of heterochromatin and inhibition of specific gene expression starts as early as EGA in the bovine, a stage when many genes are activated for expression. PRMT5 can methylate Arg-3 of H2A (in addition to Arg-8 of H3), which also leads to gene silencing^55^. This particular methylation may repress differentiation-associated genes, because PRMT5 was found to be important in early mouse embryos by maintaining pluripotency likely through the leukemia inhibitory factor (LIF)/Stat3 pathway^55^. In bovine embryos, a burst of expression in *PRMT5* was found at the 8-cell stage (Fig. 2G), when the embryos start to lose totipotency to a pluripotent state^56^. Together these data suggest that *PRMT5* may also be associated with maintaining pluripotency in the bovine.

Transcripts for the methylation maintenance enzyme, *DNMT1*, could only be observed before the 4-cell stage (Fig. 6A). This is consistent with the conclusion that the early embryos passively de-methylate due to a lack of this enzyme^57^. However, significant *DNMT1* activity was found in *in vitro* produced embryos after the 8-cell stage, suggesting abnormal maintenance of DNA methylation^58^. Interestingly, the epigenetic regulator of *DNMT1*, *UHRF1*, had the same expression pattern as *DNMT1* in our study (Table S6), suggesting the abnormal active regulation of this important DNA modifier by *in vitro* culture conditions.

In mice, the genome of the male pronucleus is actively de-methylated soon after fertilization^27^, but this genome-wide DNA de-methylation event has not be observed in sheep, and is less dramatic in cattle and pigs^59–61^. This difference may be explained by the observations that the male pronuclei of the bovine zygotes underwent de-methylation and immediately *de novo* re-methylation before the 2-cell stage^28^. Such rapid re-methylation likely requires high levels of DNA *de novo* methyl transferases. Our data, however, showed that the 2-cell bovine embryo did not contain high levels of mRNAs for either *DNMT3A* or *DNMT3B* (Fig. 6B,C). The only mRNA present for *de novo* methylation at this stage was *DNMT3B* (Fig. 6C). Albeit still low, it may be responsible for this rapid re-methylation process. DNA re-methylation at later stages up to the blastocysts as reported in IVF, parthenogenetic and SCNT embryos^58^, may be a result of *DNMT3A* as supported by our results of a dramatic increase in this transcript (Fig. 6B). In summary, bovine pre-implantation embryos’ *de novo* methylation may be primarily carried out by *DNMT3B* in early stages and then *DNMT3A* at the blastocyst stage.

Although transcripts for both *TET2* and *TET3* (Fig. 6E,F) were detected before the 8-cell stage and appeared to have been turned off by EGA, their levels in bovine early embryos were only moderate, supporting the observation that DNA de-methylation in the bovine is not be as dramatic as in the mouse^62^. The mRNA for *TET1*, however, had the opposite dynamic pattern. It was turned on by EGA and continued to increase until the 32-cell stage (Fig. 6D). Together, our data suggest that during the entire early bovine embryo development, DNA was de-methylated by a combined passive (lack of *DNMT1*) and active (presence of *TET1*) mechanisms. Because *TET3* is present in the oocytes, it is likely responsible for the de-methylation of DNA in the male pronucleus. However, the combined presence of mRNAs for *TET1-3* from oocytes to morula suggests that active de-methylation of DNA likely persists during the entire bovine pre-implantation embryo development. This, combined with the pattern of *DNMT1*, suggests that the bovine *in vivo* early embryos are de-methylated through both active and passive mechanisms from oocytes to blastocysts. Interestingly, the mRNA for the gene thymine DNA glycosylase (*TGD*), which participates in DNA demethylation by actively revers 5-hmC to cytosine through base excision repair^63^, was not detected in our database. This indicate that during early embryogenesis, active DNA demethylation can only be achieved by replication-dependent dilution of oxidized forms of 5mC.

*In vitro* cultured bovine embryos have a slower developmental speed (at least to EGA) compared to their *in vivo* counterparts as reflected by the delayed EGA to 8–16 cell stage^18^. Transcription of histone variant genes *H2AFV* and *H2AFZ*, histone methyltransferase *PRMT5*, histone demethylase *KDM5B*, and histone acetyltransferase *HAT1* started at the 4-cell stage and peaked at the 8-cell stage *in vivo*. However, these changes peaked in *in vitro* embryos at the 16-cell stage, corresponding to their EGA. Transcripts for histone acetyltransferase *KAT8* exhibited opposite dynamics. They peaked in 8-cell in *in vivo* but reached a nadir in their *in vitro* counterparts. However, neither patterns had significance differences across development. Additionally, histone deacetylases *HDAC1*, *HDAC8*, and DNA demethylase *TET1* all had significant increases at the blastocysts stage in *in vitro* embryos only, potentially contributing to the epigenetic anomalies caused by *in vitro* culture^26^.

Using real time qPCR, we randomly selected 5 genes for validation. All changes from RNA-seq were confirmed by qPCR except for *KANSL2* between 4-cell vs. blastocyst. Although high correlation between results of qPCR and RNA-Seq are expected^64–66^, this low level of inconsistency is not totally unexpected because each technology has its own strengths and weaknesses. The RNA-seq technology is highly automated for global gene expression profiling and may generate a low percentage of data that do not exactly reflect the expression status. Real time qPCR on the other hand, is more accurate but limited to quantification of one gene at a time^67^. In the case of discrepancy between results of RNA-seq and qPCR, we regard that the RNA-seq result may be inaccurate.

## Materials and Methods

### Data Mining

Previously, we established the transcriptional profiles of single bovine *in vivo* derived oocytes and pre-implantation embryos by RNA-Seq (Gene Expression Omnibus (GEO) accession number GSE59186)^18^. The quality and reproducibility of the preparation and sequencing methods were demonstrated by the high Pearson correlation efficiencies between the replicates^18^. We also downloaded RNA-Seq dataset (GEO accession number GSE52415)^17^ of *in vitro* produced oocytes and pre-implantation embryos to compare the differences between embryos developed under different environmental conditions.

Sequencing reads were normalized to TPM as final measurement of transcript abundance after trimming and mapping. TPM was preferred to FKPM because it normalizes transcriptome size across developmental stages^68^. The formula of TPM calculation for any given gene α (TPMα) is shown below: where *N*_*α*_/*L*_*α*_ means the read counts for gene α divided by its transcript length and gives transcript length-adjusted read counts; while $\sum _{i=1}^{n}\,({N}_{i}/{L}_{i})$ stands for the sum of length-adjusted read counts for all genes and is used to normalize transcriptome sizes of different samples.

$$TPM\alpha =\frac{{N}_{\alpha }/{L}_{\alpha }}{{\sum }_{i=1}^{n}\,({N}_{i}/{L}_{i})}\times {10}^{6}$$

To analyze the transcriptional profiles of epigenetic modifiers, lists of bovine annotated histone variants and histone/DNA modification proteins were generated according to the functional hierarchies in KEGG BRITE (http://www.genome.jp/kegg/brite.html#gene). Full lists of these genes are summarized in Table S8. Here, histone modifying enzymes refer to those that contain catalytic domains for post-translational modifications and/or are subunits of catalytic complexes. The transcriptional profiles for these genes were subsequently established by searching for these transcripts in our RNA-Seq dataset. Transcript abundance at each developmental stage was measured by TPM (Mean ± SEM). Genes with mean TPM values over 1, 50 and 1,000 were considered as detected, abundant and highly abundant, respectively.

### Quantitative Real-Time PCR (qRT-PCR) validation of RNA-Seq data

Our results were also validated using qRT-PCR. Three genes were randomly selected from the list for histone related genes that had significant changes across stages. These included *H3F3B* (histone variant), *SETDB1* (histone methyltransferase) and *HDAC1* (histone deacetylase). Additionally, two genes were randomly selected from outside our list of genes that changed significantly, including *KANSL2* and *ASH2L*. Their transcript abundance was measured in 4- to- 8-cell embryos and blastocysts. Expression level of each gene was normalized to *GAPDH* and inter-stage comparison was made using ∆∆Ct, which was later transformed to log_2_(fold changes). Only a difference of 2-fold change or Log_2_ (fold change) of greater than 1 was regarded as significantly changed.

### Statistical analysis

All statistical analyses were conducted in Minitab (Minitab 18 Statistical Software, 2010; www.minitab.com), and significance was defined as p < 0.05. One-way ANOVA was used for comparing transcript abundance across stages. Multiple comparisons with Fisher’s Least Significant Difference (LSD) was used for pair-wise comparisons.

## Supplementary information

supplementary tables
