# Supplementary material for: Analysis of mRNA abundance for histone variants, histone- and DNA-modifiers in bovine in vivo and in vitro oocytes and embryos
Source: Sci Rep. 2019 Feb 4;9:1217. doi: 10.1038/s41598-018-38083-4 (PMC6362035; doi:10.1038/s41598-018-38083-4)
Supplement: Supplementary file 1 — supplementary tables [file 41598_2018_38083_MOESM1_ESM.docx]

**Analysis of mRNA abundance for histone variants, histone- and DNA-modifiers in bovine *in vivo* and *in vitro* oocytes and embryos**

Duan J^1*^, Zhu L^1*^, Dong H^2^, Zheng X^2^, Jiang Z^1,3^, Chen J^2^, Tian XC^1^ ^1^Department of Animal Science, University of Connecticut, Storrs, CT, 06269, USA; ^2^Xinjiang Academy of Animal Science, Urumqi, Xinjiang, PR China;

^3^Current address: School of Animal Sciences, Louisiana State University, Baton Rouge, LA, USA

Jingyue (Ellie) Duan (jingyue.duan@uconn.edu)

Linkai Zhu (Linkai.zhu@uconn.edu)

Hong Dong (595796086@qq.com)

Xinbao Zheng (zhaogm1972@126.com)

Zongliang Jiang (zjiang@agcenter.lsu.edu)

Jingbo Chen (chenjb126@126.com)

Xiuchun (Cindy) Tian (xiuchun.tian@uconn.edu)

^*^These authors contributed equally to this work.

Corresponding authors:

Xiuchun (Cindy) Tian, PhD, Center for Regenerative Biology, Department of Animal Science, University of Connecticut, 1390 Storrs Rd, Storrs, CT 06269, USA. Phone: (860) 486-9087, Email: xiuchun.tian@uconn.edu.

Jingbo Chen, PhD, Institute of Animal Science, Xinjiang Academy of Animal Science, 151 East Kalamay Street, Urumqi, Xinjiang, PR China, 830000. Phone: +86 0991-4843303, Fax: +86 0991-4843303, Email: chenjb126@126.com

**Supplementary Information**

Table S1. Transcript abundance of bovine histone variants (Mean TPM).

|  | **Mature** | **2-Cell** | **4-Cell** | **8-Cell** | **16-Cell** | **32-Cell** | **Compact** | **Blastocyst** |
| --- | --- | --- | --- | --- | --- | --- | --- | --- |
|  | **oocyte** |  |  |  |  |  | **morula** |  |
|  |  |  |  |  |  |  |  |  |
| *H1F0* | 0.0 | 0.7 | 0.4 | 0.1 | 0.6 | 0.2 | 0.1 | 0.1 |
|  |  |  |  |  |  |  |  |  |
| *H1FNT* | 0.2 | 0.0 | 0.7 | 0.0 | 0.0 | 0.0 | 0.0 | 0.0 |
|  |  |  |  |  |  |  |  |  |
| *H1FOO* | 1872.3 | 1016.9 | 708.3 | 32.9 | 0.2 | 0.3 | 1.4 | 0.2 |
|  |  |  |  |  |  |  |  |  |
| *H1FX* | 0.5 | 0.0 | 0.3 | 0.0 | 0.0 | 0.0 | 0.0 | 0.4 |
|  |  |  |  |  |  |  |  |  |
| *H2AFJ* | 0.0 | 0.6 | 0.0 | 0.0 | 1.9 | 0.2 | 0.0 | 4.7 |
|  |  |  |  |  |  |  |  |  |
| *H2AFV* | 5.3 | 14.3 | 22.0 | 168.2 | 173.6 | 266.7 | 276.3 | 153.9 |
|  |  |  |  |  |  |  |  |  |
| *H2AFX* | 9.5 | 14.6 | 8.8 | 6.1 | 9.7 | 7.0 | 8.3 | 6.7 |
|  |  |  |  |  |  |  |  |  |
| *H2AFY* | 5.4 | 2.1 | 3.2 | 9.9 | 3.0 | 6.2 | 4.1 | 11.4 |
|  |  |  |  |  |  |  |  |  |
| *H2AFY2* | 0.0 | 0.1 | 0.0 | 0.0 | 0.0 | 0.0 | 0.4 | 0.7 |
|  |  |  |  |  |  |  |  |  |
| *H2AFZ* | 2.9 | 6.8 | 9.9 | 769.3 | 723.9 | 535.1 | 632.3 | 254.7 |
|  |  |  |  |  |  |  |  |  |
| *CENPA* | 61.0 | 11.9 | 27.2 | 20.1 | 29.8 | 149.7 | 44.1 | 5.3 |
|  |  |  |  |  |  |  |  |  |
| *H3F3A* | 11879.3 | 3059.0 | 2293.1 | 4462.0 | 3943.9 | 4115.5 | 3470.1 | 3980.9 |
|  |  |  |  |  |  |  |  |  |
| *H3F3B* | 4220.1 | 4987.6 | 2344.6 | 1689.4 | 3041.6 | 2122.0 | 1209.5 | 639.1 |
|  |  |  |  |  |  |  |  |  |
| *H3F3C* | 13.3 | 10.5 | 9.9 | 5.6 | 36.6 | 8.2 | 4.7 | 10.7 |
|  |  |  |  |  |  |  |  |  |

Table S2. Transcript abundance of bovine histone methyltransferases (Mean TPM).

|  | **Mature** | **2-Cell** | **4-Cell** | **8-Cell** | **16-Cell** | **32-Cell** | **Compact** | **Blastocyst** |
| --- | --- | --- | --- | --- | --- | --- | --- | --- |
|  | **oocyte** |  |  |  |  |  | **morula** |  |
|  |  |  |  |  |  |  |  |  |
| *ASH1L* | 1.7 | 1.1 | 2.4 | 1.0 | 5.1 | 0.8 | 0.7 | 0.2 |
|  |  |  |  |  |  |  |  |  |
| *CARM1* | 4.6 | 0.1 | 12.4 | 0.7 | 18.5 | 3.5 | 2.9 | 0.3 |
|  |  |  |  |  |  |  |  |  |
| *DOT1L* | 0.4 | 1.5 | 3.9 | 2.1 | 3.9 | 2.4 | 1.6 | 2.9 |
|  |  |  |  |  |  |  |  |  |
| *EZH1* | 0.1 | 0.0 | 0.6 | 0.0 | 1.1 | 0.0 | 0.1 | 0.0 |
|  |  |  |  |  |  |  |  |  |
| *EZH2* | 36.1 | 88.7 | 243.5 | 123.0 | 63.2 | 76.6 | 68.5 | 24.3 |
|  |  |  |  |  |  |  |  |  |
| *KMT2A* | 0.3 | 0.2 | 0.4 | 0.2 | 1.8 | 0.1 | 0.6 | 0.4 |
|  |  |  |  |  |  |  |  |  |
| *KMT2B* | 2.6 | 0.1 | 8.2 | 0.3 | 12.2 | 0.8 | 0.6 | 0.3 |
|  |  |  |  |  |  |  |  |  |
| *KMT2C* | 33.8 | 7.1 | 23.5 | 4.9 | 28.5 | 4.2 | 5.3 | 3.8 |
|  |  |  |  |  |  |  |  |  |
| *KMT2D* | 0.2 | 0.0 | 0.3 | 0.1 | 0.3 | 0.2 | 0.1 | 0.0 |
|  |  |  |  |  |  |  |  |  |
| *KMT2E* | 18.2 | 1.8 | 38.1 | 1.7 | 42.5 | 1.3 | 1.1 | 1.1 |
|  |  |  |  |  |  |  |  |  |
| *PRDM2* | 1.2 | 0.3 | 1.2 | 0.4 | 1.9 | 1.1 | 0.4 | 0.6 |
|  |  |  |  |  |  |  |  |  |
| *PRDM6* | 0.5 | 0.0 | 0.3 | 0.0 | 1.7 | 0.1 | 0.0 | 0.0 |
|  |  |  |  |  |  |  |  |  |
| *PRDM7* | 0.0 | 0.0 | 0.0 | 0.0 | 0.0 | 0.0 | 0.0 | 0.0 |
|  |  |  |  |  |  |  |  |  |
| *PRDM8* | 0.0 | 0.0 | 0.0 | 0.0 | 0.1 | 1.1 | 0.0 | 0.0 |
|  |  |  |  |  |  |  |  |  |
| *PRDM9* | 0.0 | 0.0 | 0.0 | 0.0 | 0.0 | 0.0 | 0.0 | 0.0 |
|  |  |  |  |  |  |  |  |  |
| *PRMT1* | 79.8 | 10.3 | 87.9 | 8.1 | 110.2 | 50.2 | 7.0 | 16.3 |
|  |  |  |  |  |  |  |  |  |
| *PRMT2* | 2.7 | 1.3 | 6.6 | 2.9 | 5.5 | 4.5 | 3.0 | 1.4 |
|  |  |  |  |  |  |  |  |  |
| *PRMT3* | 7.9 | 14.3 | 9.8 | 12.7 | 16.8 | 5.0 | 2.9 | 6.6 |
|  |  |  |  |  |  |  |  |  |
| *PRMT5* | 8.1 | 38.8 | 46.3 | 85.2 | 71.6 | 270.0 | 28.3 | 53.9 |
|  |  |  |  |  |  |  |  |  |
| *PRMT6* | 0.0 | 0.1 | 0.3 | 0.7 | 1.2 | 0.3 | 0.5 | 0.5 |
|  |  |  |  |  |  |  |  |  |
| *PRMT7* | 0.1 | 9.0 | 5.5 | 15.6 | 8.4 | 38.7 | 25.6 | 21.4 |
|  |  |  |  |  |  |  |  |  |
| *PRMT8* | 0.0 | 0.0 | 0.0 | 0.0 | 0.0 | 0.0 | 0.3 | 0.0 |
|  |  |  |  |  |  |  |  |  |
| *PRMT9* | 9.6 | 3.3 | 8.0 | 3.1 | 5.3 | 3.0 | 1.2 | 1.8 |
|  |  |  |  |  |  |  |  |  |
| *SETD2* | 0.1 | 2.8 | 0.3 | 2.3 | 0.7 | 2.5 | 0.7 | 1.8 |
|  |  |  |  |  |  |  |  |  |
| *SETD3* | 73.1 | 17.9 | 47.0 | 18.3 | 27.0 | 11.9 | 30.6 | 14.6 |
|  |  |  |  |  |  |  |  |  |
| *SETD6* | 3.1 | 13.8 | 11.7 | 7.3 | 9.2 | 2.1 | 17.3 | 16.1 |
|  |  |  |  |  |  |  |  |  |
| *SETD7* | 0.0 | 0.9 | 0.0 | 0.3 | 0.0 | 0.1 | 0.7 | 0.2 |
|  |  |  |  |  |  |  |  |  |
| *SETD8* | 3.7 | 1.4 | 3.3 | 2.2 | 4.6 | 1.7 | 1.1 | 1.7 |
|  |  |  |  |  |  |  |  |  |
| *SETDB1* | 15.4 | 0.9 | 46.4 | 2.2 | 72.3 | 11.2 | 1.2 | 0.9 |
|  |  |  |  |  |  |  |  |  |
| *SETDB2* | 26.4 | 34.4 | 19.1 | 39.3 | 17.5 | 25.4 | 8.6 | 40.4 |
|  |  |  |  |  |  |  |  |  |
| *SETMAR* | 0.1 | 0.9 | 0.2 | 0.2 | 0.0 | 0.4 | 0.0 | 0.2 |
|  |  |  |  |  |  |  |  |  |
| *ZNF488* | 0.0 | 1.1 | 0.2 | 0.3 | 0.0 | 0.0 | 0.0 | 0.2 |
|  |  |  |  |  |  |  |  |  |
| *EHMT1* | 0.9 | 7.8 | 11.4 | 0.7 | 0.8 | 0.7 | 0.4 | 1.1 |
|  |  |  |  |  |  |  |  |  |
| *EHMT2* | 2.1 | 3.8 | 7.3 | 1.8 | 0.1 | 0.2 | 0.1 | 0.4 |
|  |  |  |  |  |  |  |  |  |
| *LOC540197* | 0.3 | 1.2 | 2.1 | 1.7 | 1.1 | 2.3 | 1.7 | 1.7 |
|  |  |  |  |  |  |  |  |  |
| *WHSC1* | 1.8 | 15.5 | 8.1 | 1.1 | 0.4 | 1.0 | 1.3 | 3.7 |
|  |  |  |  |  |  |  |  |  |
| *WHSC1L1* | 2.3 | 11.7 | 13.1 | 0.9 | 0.4 | 1.4 | 1.0 | 1.2 |
|  |  |  |  |  |  |  |  |  |
| *LOC520584* | 0.0 | 0.0 | 0.0 | 0.0 | 0.9 | 0.5 | 0.0 | 0.0 |
|  |  |  |  |  |  |  |  |  |
| *SETD1A* | 0.9 | 1.5 | 1.4 | 0.3 | 0.2 | 0.2 | 0.3 | 0.2 |
|  |  |  |  |  |  |  |  |  |
| *SETD1B* | 0.5 | 1.6 | 2.0 | 0.5 | 1.2 | 0.5 | 0.3 | 1.0 |
|  |  |  |  |  |  |  |  |  |

| *LOC534913* | 56.7 | 51.0 | 59.8 | 7.0 | 27.5 | 15.0 | 15.0 | 40.5 |
| --- | --- | --- | --- | --- | --- | --- | --- | --- |
|  |  |  |  |  |  |  |  |  |
| *SMYD1* | 0.0 | 0.0 | 0.0 | 0.0 | 0.0 | 0.0 | 0.0 | 0.0 |
|  |  |  |  |  |  |  |  |  |
| *SMYD2* | 3.1 | 30.5 | 42.9 | 3.6 | 4.4 | 4.2 | 4.3 | 4.2 |
|  |  |  |  |  |  |  |  |  |
| *SMYD3* | 1.4 | 7.2 | 9.0 | 1.1 | 2.2 | 2.2 | 1.0 | 4.0 |
|  |  |  |  |  |  |  |  |  |
| *SUV39H1* | 0.0 | 4.4 | 2.5 | 1.5 | 0.2 | 0.8 | 0.2 | 0.2 |
|  |  |  |  |  |  |  |  |  |
| *SUV39H2* | 12.9 | 61.9 | 129.9 | 5.9 | 3.5 | 1.1 | 0.2 | 2.2 |
|  |  |  |  |  |  |  |  |  |
| *SUV420H1* | 85.8 | 95.9 | 99.3 | 8.4 | 6.7 | 3.2 | 3.1 | 4.0 |
|  |  |  |  |  |  |  |  |  |
| *SUV420H2* | 1.7 | 2.5 | 1.9 | 0.9 | 0.0 | 0.0 | 0.0 | 0.0 |
|  |  |  |  |  |  |  |  |  |

Table S3. Transcript abundance of bovine histone demethylases (Mean TPM).

|  | **Mature** | **2-Cell** | **4-Cell** | **8-Cell** | **16-Cell** | **32-Cell** | **Compact** | **Blastocyst** |
| --- | --- | --- | --- | --- | --- | --- | --- | --- |
|  | **oocyte** |  |  |  |  |  | **morula** |  |
|  |  |  |  |  |  |  |  |  |
| *JARID2* | 79.7 | 33.3 | 32.0 | 30.2 | 24.6 | 15.0 | 30.5 | 20.9 |
|  |  |  |  |  |  |  |  |  |
| *JMJD1C* | 6.5 | 3.0 | 6.7 | 1.0 | 7.9 | 2.0 | 0.9 | 0.5 |
|  |  |  |  |  |  |  |  |  |
| *JMJD6* | 3.2 | 2.3 | 1.2 | 3.7 | 3.0 | 11.3 | 1.7 | 3.8 |
|  |  |  |  |  |  |  |  |  |
| *JMJD7* | 0.2 | 0.1 | 1.6 | 1.9 | 0.9 | 0.7 | 3.0 | 0.3 |
|  |  |  |  |  |  |  |  |  |
| *KDM1A* | 152.1 | 61.5 | 132.9 | 87.5 | 87.6 | 112.5 | 33.2 | 61.0 |
|  |  |  |  |  |  |  |  |  |
| *KDM1B* | 201.9 | 12.9 | 246.7 | 3.0 | 84.6 | 3.6 | 1.0 | 2.6 |
|  |  |  |  |  |  |  |  |  |
| *KDM2A* | 1.0 | 4.6 | 2.4 | 4.7 | 8.9 | 1.8 | 1.7 | 4.0 |
|  |  |  |  |  |  |  |  |  |
| *KDM2B* | 0.5 | 12.0 | 2.3 | 7.5 | 2.9 | 2.6 | 9.2 | 8.7 |
|  |  |  |  |  |  |  |  |  |
| *KDM6A* | 1.0 | 9.6 | 1.2 | 3.1 | 0.9 | 5.0 | 1.1 | 1.6 |
|  |  |  |  |  |  |  |  |  |
| *KDM6B* | 0.7 | 0.0 | 0.7 | 0.1 | 1.8 | 0.0 | 0.5 | 0.2 |
|  |  |  |  |  |  |  |  |  |
| *KDM7A* | 2.0 | 0.2 | 3.2 | 0.1 | 4.8 | 0.1 | 0.1 | 0.1 |
|  |  |  |  |  |  |  |  |  |
| *KDM8* | 0.1 | 0.1 | 0.0 | 0.2 | 0.0 | 0.0 | 0.0 | 0.1 |
|  |  |  |  |  |  |  |  |  |
| *PHF2* | 0.9 | 0.7 | 0.0 | 0.1 | 0.6 | 0.1 | 0.2 | 0.1 |
|  |  |  |  |  |  |  |  |  |
| *PHF8* | 3.2 | 2.2 | 1.5 | 0.7 | 6.5 | 2.2 | 2.0 | 0.8 |
|  |  |  |  |  |  |  |  |  |
| *UTY* | 0.2 | 0.1 | 0.3 | 0.0 | 0.1 | 0.0 | 2.0 | 1.3 |
|  |  |  |  |  |  |  |  |  |
| *KDM3A* | 22.9 | 16.7 | 6.3 | 13.5 | 13.9 | 6.9 | 5.7 | 7.0 |
|  |  |  |  |  |  |  |  |  |
| *KDM3B* | 0.2 | 2.5 | 2.4 | 0.3 | 0.2 | 0.3 | 0.3 | 1.5 |
|  |  |  |  |  |  |  |  |  |
| *KDM4A* | 1.4 | 9.9 | 14.2 | 7.6 | 10.5 | 9.2 | 6.7 | 4.4 |
|  |  |  |  |  |  |  |  |  |
| *KDM4B* | 0.0 | 2.9 | 1.8 | 0.3 | 0.2 | 0.0 | 0.1 | 0.3 |
|  |  |  |  |  |  |  |  |  |
| *KDM4C* | 4.3 | 11.2 | 9.4 | 0.8 | 1.5 | 1.3 | 1.4 | 0.8 |
|  |  |  |  |  |  |  |  |  |
| *KDM5A* | 23.4 | 15.9 | 38.3 | 5.7 | 14.0 | 8.5 | 5.1 | 10.1 |
|  |  |  |  |  |  |  |  |  |
| *KDM5B* | 0.3 | 1.8 | 2.9 | 171.1 | 54.4 | 144.0 | 59.6 | 43.0 |
|  |  |  |  |  |  |  |  |  |
| *KDM5C* | 0.1 | 1.4 | 3.5 | 0.8 | 1.2 | 0.6 | 0.4 | 0.5 |
|  |  |  |  |  |  |  |  |  |
| *LOC617971* | 0.0 | 0.0 | 0.0 | 0.0 | 0.1 | 0.2 | 0.0 | 0.0 |
|  |  |  |  |  |  |  |  |  |
| *LOC524768* | 0.0 | 0.0 | 0.0 | 2.5 | 0.2 | 0.7 | 0.0 | 0.0 |
|  |  |  |  |  |  |  |  |  |
| *HR* | 0.0 | 0.0 | 0.0 | 0.0 | 0.0 | 0.0 | 0.0 | 0.0 |
|  |  |  |  |  |  |  |  |  |
| *C10H14orf169* | 4.9 | 5.0 | 5.6 | 7.1 | 17.4 | 14.1 | 13.9 | 7.8 |
|  |  |  |  |  |  |  |  |  |
| *MINA* | 72.9 | 150.7 | 254.0 | 32.3 | 27.4 | 24.4 | 19.0 | 20.6 |
|  |  |  |  |  |  |  |  |  |

Table S4. Transcript abundance of bovine histone acetyltransferases (Mean TPM).

|  | **Mature** | **2-Cell** | **4-Cell** | **8-Cell** | **16-Cell** | **32-Cell** | **Compact** | **Blastocyst** |
| --- | --- | --- | --- | --- | --- | --- | --- | --- |
|  | **oocyte** |  |  |  |  |  | **morula** |  |
|  |  |  |  |  |  |  |  |  |
| *CLOCK* | 11.8 | 1.0 | 56.2 | 1.6 | 52.9 | 19.0 | 0.4 | 0.2 |
|  |  |  |  |  |  |  |  |  |
| *CREBBP* | 43.5 | 16.9 | 37.9 | 13.6 | 52.5 | 12.7 | 6.9 | 8.8 |
|  |  |  |  |  |  |  |  |  |
| *ELP3* | 0.3 | 4.0 | 5.9 | 2.4 | 2.1 | 4.8 | 1.8 | 1.7 |
|  |  |  |  |  |  |  |  |  |
| *GTF3C4* | 0.0 | 0.2 | 0.4 | 0.1 | 2.6 | 0.4 | 0.0 | 0.0 |
|  |  |  |  |  |  |  |  |  |
| *HAT1* | 6.7 | 119.3 | 30.8 | 110.8 | 51.2 | 129.8 | 46.5 | 72.7 |
|  |  |  |  |  |  |  |  |  |
| *KAT2A* | 6.8 | 0.3 | 14.2 | 0.9 | 14.0 | 3.6 | 0.7 | 1.0 |
|  |  |  |  |  |  |  |  |  |
| *KAT2B* | 0.5 | 6.8 | 1.8 | 8.8 | 1.2 | 3.6 | 2.8 | 5.1 |
|  |  |  |  |  |  |  |  |  |
| *KAT5* | 0.3 | 3.1 | 16.0 | 5.2 | 34.5 | 8.6 | 4.0 | 2.7 |
|  |  |  |  |  |  |  |  |  |
| *KAT6A* | 3.6 | 0.6 | 5.5 | 0.3 | 8.4 | 2.2 | 0.3 | 0.2 |
|  |  |  |  |  |  |  |  |  |
| *KAT6B* | 14.0 | 1.0 | 25.9 | 0.7 | 22.6 | 1.4 | 3.5 | 1.6 |
|  |  |  |  |  |  |  |  |  |
| *KAT7* | 5.7 | 7.9 | 32.6 | 8.3 | 43.8 | 7.0 | 3.5 | 7.6 |
|  |  |  |  |  |  |  |  |  |
| *KAT8* | 21.4 | 94.2 | 67.7 | 72.9 | 70.4 | 161.4 | 63.2 | 96.1 |
|  |  |  |  |  |  |  |  |  |
| *NAA40* | 0.8 | 3.4 | 3.2 | 10.3 | 3.6 | 6.8 | 7.2 | 5.4 |
|  |  |  |  |  |  |  |  |  |
| *NAA60* | 0.0 | 0.8 | 7.0 | 1.2 | 6.2 | 1.0 | 2.7 | 0.6 |
|  |  |  |  |  |  |  |  |  |
| *NCOA1* | 44.1 | 5.5 | 27.5 | 5.6 | 19.9 | 7.9 | 11.1 | 8.4 |
|  |  |  |  |  |  |  |  |  |
| *NCOA2* | 79.5 | 4.7 | 66.2 | 2.2 | 59.2 | 4.1 | 1.1 | 1.3 |
|  |  |  |  |  |  |  |  |  |
| *NCOA3* | 4.1 | 2.2 | 16.7 | 2.3 | 16.9 | 4.4 | 3.8 | 2.6 |
|  |  |  |  |  |  |  |  |  |
| *TAF1* | 2.0 | 3.1 | 6.7 | 5.3 | 4.3 | 3.2 | 1.3 | 2.1 |
|  |  |  |  |  |  |  |  |  |
| *CDYL* | 32.9 | 31.3 | 36.5 | 5.4 | 3.7 | 3.2 | 3.5 | 1.3 |
|  |  |  |  |  |  |  |  |  |
| *CDYL2* | 0.0 | 0.3 | 0.1 | 0.0 | 0.0 | 0.0 | 0.0 | 0.1 |
|  |  |  |  |  |  |  |  |  |

Table S5. Transcript abundance of bovine histone deacetylases (Mean TPM).

|  | **Mature** | **2-Cell** | **4-Cell** | **8-Cell** | **16-Cell** | **32-Cell** | **Compact** | **Blastocyst** |
| --- | --- | --- | --- | --- | --- | --- | --- | --- |
|  | **oocyte** |  |  |  |  |  | **morula** |  |
|  |  |  |  |  |  |  |  |  |
| *HDAC1* | 71.8 | 185.1 | 88.6 | 95.4 | 100.0 | 161.3 | 112.4 | 123.4 |
|  |  |  |  |  |  |  |  |  |
| *HDAC2* | 3.8 | 33.7 | 23.0 | 30.6 | 60.9 | 15.9 | 9.0 | 27.0 |
|  |  |  |  |  |  |  |  |  |
| *HDAC3* | 82.7 | 8.5 | 38.0 | 4.5 | 16.4 | 11.4 | 5.2 | 6.8 |
|  |  |  |  |  |  |  |  |  |
| *HDAC4* | 0.0 | 0.1 | 0.0 | 0.0 | 0.0 | 0.0 | 0.2 | 0.1 |
|  |  |  |  |  |  |  |  |  |
| *HDAC5* | 0.3 | 6.7 | 0.8 | 9.0 | 1.3 | 13.8 | 4.6 | 6.7 |
|  |  |  |  |  |  |  |  |  |
| *HDAC6* | 0.0 | 8.8 | 2.4 | 4.6 | 3.3 | 18.3 | 11.1 | 5.2 |
|  |  |  |  |  |  |  |  |  |
| *HDAC7* | 1.7 | 2.5 | 7.6 | 0.7 | 16.4 | 1.6 | 1.1 | 0.9 |
|  |  |  |  |  |  |  |  |  |
| *HDAC8* | 61.3 | 66.5 | 25.6 | 22.1 | 17.7 | 64.5 | 47.2 | 32.7 |
|  |  |  |  |  |  |  |  |  |
| *HDAC9* | 7.0 | 0.1 | 5.2 | 0.0 | 3.6 | 0.6 | 0.0 | 0.0 |
|  |  |  |  |  |  |  |  |  |
| *SIRT1* | 7.2 | 29.2 | 17.2 | 11.5 | 15.2 | 5.5 | 6.8 | 8.1 |
|  |  |  |  |  |  |  |  |  |
| *SIRT2* | 0.0 | 0.2 | 0.0 | 0.1 | 1.1 | 1.0 | 2.1 | 0.5 |
|  |  |  |  |  |  |  |  |  |
| *SIRT3* | 54.7 | 3.0 | 32.1 | 1.7 | 19.9 | 2.8 | 3.9 | 0.0 |
|  |  |  |  |  |  |  |  |  |
| *SIRT4* | 0.0 | 1.2 | 0.9 | 3.8 | 0.4 | 2.0 | 0.7 | 0.9 |
|  |  |  |  |  |  |  |  |  |
| *SIRT5* | 5.5 | 4.3 | 5.8 | 2.9 | 19.7 | 3.4 | 9.5 | 2.2 |
|  |  |  |  |  |  |  |  |  |
| *SIRT6* | 0.0 | 0.0 | 0.0 | 0.2 | 0.6 | 0.0 | 1.0 | 0.2 |
|  |  |  |  |  |  |  |  |  |
| *SIRT7* | 0.9 | 4.4 | 0.8 | 2.0 | 1.7 | 5.7 | 6.3 | 2.3 |
|  |  |  |  |  |  |  |  |  |
| *LOC100849030* | 9.0 | 6.0 | 8.1 | 1.6 | 0.2 | 0.0 | 0.0 | 0.2 |
|  |  |  |  |  |  |  |  |  |
| *HDAC10* | 0.5 | 5.5 | 3.1 | 1.4 | 0.1 | 0.3 | 0.1 | 0.3 |
|  |  |  |  |  |  |  |  |  |
| *HDAC11* | 0.9 | 4.7 | 5.9 | 2.2 | 0.0 | 0.0 | 0.0 | 0.0 |
|  |  |  |  |  |  |  |  |  |

Table S6. Transcript abundance of bovine DNA modifiers (Mean TPM).

|  | **Mature** | **2-Cell** | **4-Cell** | **8-Cell** | **16-Cell** | **32-Cell** | **Compact** | **Blastocyst** |
| --- | --- | --- | --- | --- | --- | --- | --- | --- |
|  | **oocyte** |  |  |  |  |  | **morula** |  |
|  |  |  |  |  |  |  |  |  |
| *DNMT1* | 135.3 | 129.3 | 103.0 | 5.6 | 5.6 | 4.9 | 3.6 | 2.3 |
|  |  |  |  |  |  |  |  |  |
| *DNMT3A* | 0.9 | 0.9 | 2.4 | 0.7 | 10.0 | 0.7 | 3.3 | 29.9 |
|  |  |  |  |  |  |  |  |  |
| *DNMT3B* | 5.7 | 18.0 | 35.9 | 5.6 | 1.6 | 0.1 | 0.3 | 6.8 |
|  |  |  |  |  |  |  |  |  |
| *DNMT3L* | 0.0 | 0.0 | 0.0 | 0.0 | 0.1 | 0.0 | 0.0 | 0.0 |
|  |  |  |  |  |  |  |  |  |
| *TET1* | 0.0 | 0.1 | 0.0 | 2.1 | 5.5 | 6.7 | 2.7 | 2.9 |
|  |  |  |  |  |  |  |  |  |
| *TET2* | 0.6 | 2.0 | 2.3 | 0.3 | 0.0 | 0.0 | 0.0 | 0.0 |
|  |  |  |  |  |  |  |  |  |
| *TET3* | 5.3 | 7.5 | 6.0 | 0.6 | 0.0 | 0.0 | 0.0 | 0.2 |
|  |  |  |  |  |  |  |  |  |
| *UHRF1* | 1373.7 | 349.2 | 284.7 | 29.9 | 1.4 | 0.5 | 1.2 | 1.5 |
|  |  |  |  |  |  |  |  |  |

Table S7. Transcript abundance of bovine histone variants and histone/DNA modification proteins from *in vitro* produced embryos (Mean TPM).

| **Histone variants** | **Mature oocyte** | **4C** | **8C** | **16C** | **BL** |
| --- | --- | --- | --- | --- | --- |
| *H1FOO* | 1252.6 | 1023.9 | 593.5 | 38.3 | 2.0 |
| *H2AFV* | 39.6 | 29.9 | 247.9 | 454.8 | 312.3 |
| *H2AFX* | 862.5 | 713.5 | 490.6 | 416.9 | 107.3 |
| *H2AFY* | 6.6 | 8.0 | 94.8 | 175.4 | 24.1 |
| *H2AFZ* | 21.9 | 11.5 | 3932.6 | 3801.8 | 1194.0 |
| *CENPA* | 55.0 | 63.0 | 101.7 | 165.3 | 114.7 |
| *H3F3A* | 1129.7 | 420.0 | 1127.8 | 1836.6 | 1048.7 |
| *H3F3B* | 1384.1 | 1260.7 | 1517.1 | 1232.5 | 462.0 |
| *H3F3C* | 6.0 | 5.9 | 8.2 | 4.6 | 4.6 |
|  | | | | | |
| **Methyltransferases** | **Mature oocyte** | **4C** | **8C** | **16C** | **BL** |
| *EZH2* | 92.3 | 168.1 | 158.6 | 224.4 | 92.5 |
| *PRMT1* | 69.4 | 75.9 | 72.1 | 77.1 | 65.3 |
| *PRMT5* | 43.5 | 76.0 | 110.9 | 229.3 | 120.8 |
| *SETD3* | 84.6 | 44.1 | 20.3 | 41.1 | 45.7 |
| *SETDB1* | 76.1 | 104.4 | 106.3 | 42.9 | 13.1 |
| *LOC534913* | 76.4 | 86.5 | 52.0 | 27.5 | 71.4 |
| *SUV39H2* | 142.0 | 156.7 | 164.1 | 18.0 | 14.1 |
| *SUV420H1* | 204.5 | 311.0 | 255.7 | 59.4 | 40.8 |
|  | | | | | |
| **De-methylases** | **Mature oocyte** | **4C** | **8C** | **16C** | **BL** |
| *KDM1A* | 175.5 | 225.2 | 59.6 | 177.6 | 211.3 |
| *KDM1B* | 57.1 | 75.6 | 40.9 | 8.7 | 3.9 |
| *KDM5B* | 2.1 | 2.2 | 27.4 | 87.6 | 70.0 |
| *MINA* | 62.2 | 75.6 | 58.7 | 27.1 | 22.2 |
|  | | | | | |
| **Acetyltrasferases** | **Mature oocyte** | **4C** | **8C** | **16C** | **BL** |
| *CLOCK* | 193.8 | 285.8 | 298.6 | 106.7 | 20.7 |
| *CREBBP* | 137.8 | 171.3 | 126.3 | 51.4 | 53.5 |
| *HAT1* | 192.2 | 113.7 | 277.3 | 604.1 | 207.3 |
| *KAT8* | 53.4 | 55.6 | 24.3 | 51.4 | 59.3 |
| *NCOA2* | 185.2 | 192.9 | 159.7 | 38.6 | 36.9 |
|  | | | | | |
| **De-acetylases** | **Mature oocyte** | **4C** | **8C** | **16C** | **BL** |
| *HDAC1* | 41.4 | 77.6 | 47.9 | 175.6 | 386.3 |
| *HDAC2* | 57.0 | 62.6 | 116.0 | 216.1 | 127.1 |
| *HDAC3* | 85.4 | 44.4 | 14.4 | 15.1 | 29.1 |
| *HDAC8* | 11.3 | 26.7 | 2.1 | 7.8 | 57.8 |
| *SIRT3* | 10.6 | 27.4 | 9.6 | 1.0 | 2.3 |
|  | | | | | |
| **DNA methyltransferases** | **Mature oocyte** | **4C** | **8C** | **16C** | **BL** |
| *DNMT1* | 255.5 | 265.0 | 123.1 | 34.6 | 19.8 |
| *DNMT3A* | 4.8 | 8.5 | 4.2 | 1.0 | 72.7 |
| *DNMT3B* | 68.8 | 80.9 | 32.0 | 10.0 | 111.8 |
|  | | | | | |
| **DNA De-methylases** | **Mature oocyte** | **4C** | **8C** | **16C** | **BL** |
| *TET1* | 0.5 | 1.1 | 44.5 | 161.1 | 248.0 |
| *TET2* | 48.9 | 71.8 | 82.7 | 21.1 | 15.9 |
| *TET3* | 91.4 | 104.9 | 78.4 | 41.6 | 11.1 |

Table S8. List of KEGG bovine annotated histone variants and histone/DNA modifying proteins.

**Histone Variants**

H1F0; H1 histone family, member 0

H1FNT; H1 histone family, member N, testis-specific

H1FOO; H1 histone family, member O, oocyte-specific

H1FX; H1 histone family, member X

H2AFJ; H2A histone family, member J

H2AFV; H2A histone family, mmeber V

H2AFX; H2A histone family, member X

H2AFY (macroH2A.1); H2A histone family, member Y

H2AFY2 (macroH2A.2); H2A histone family, member Y2

H2AFZ; H2A histone family, member Z

CENPA; centromere protein A

H3F3A; H3 histone, family 3A

H3F3B; H3 histone, family 3B

H3F3C; H3 histone, family 3C

**Histone methyltransferases**

ASH1L; ASH1 like histone lysine methyltransferase

DOT1L; DOT1 like histone lysine methyltransferase

EHMT2; euchromatic histone lysine methyltransferase 2

EHMT1; euchromatic histone lysine methyltransferase 1

EZH1; enhancer of zeste 1 polycomb repressive complex 2 subunit

EZH2; enhancer of zeste 2 polycomb repressive complex 2 subunit

KMT2A; lysine methyltransferase 2A

KMT2D; lysine methyltransferase 2D

KMT2C; lysine methyltransferase 2C

KMT2B; lysine methyltransferase 2B

KMT2E; lysine methyltransferase 2E

NSD1 (LOC540197); histone-lysine N-methyltransferase, H3 lysine-36 and H4 lysine-20 specific NSD2(WHSC1); nuclear receptor binding SET domain protein 2

NSD3 (WHSC1L1); nuclear receptor binding SET domain protein 3

PRDM2; PR/SET domain 2

PRDM6; PR/SET domain 6

histone-lysine N-methyltransferase PRDM9-like

histone-lysine N-methyltransferase PRDM9-like

PRDM9; PR domain containing 9

probable histone-lysine N-methyltransferase PRDM7 PRDM7; PR domain containing 7 PR domain containing 7-like

PRDM8; PR/SET domain 8

ZNF488; zinc finger protein 488

SETD1B; SET domain containing 1B

SETD1A; SET domain containing 1A

SETD2; SET domain containing 2

SETD3; SET domain containing 3

SETD6; SET domain containing 6

SETD7; SET domain containing lysine methyltransferase 7

KMT5A (SETD8); lysine methyltransferase 5A

SETDB1; SET domain bifurcated 1

SETDB2; SET domain bifurcated 2

LOC534913; mariner-like transposase

SETMAR; SET domain without mariner transposase fusion

SMYD1; SET and MYND domain containing 1

SMYD2; SET and MYND domain containing 2

SMYD3; SET and MYND domain containing 3

SUV39H1; suppressor of variegation 3-9 homolog 1

SUV39H2; suppressor of variegation 3-9 homolog 2

KMT5C (SUV420H2); lysine methyltransferase 5C

KMT5B (SUV420H1); lysine methyltransferase 5B

PRMT1; protein arginine methyltransferase 1

PRMT2; protein arginine methyltransferase 2

PRMT3; protein arginine methyltransferase 3

CARM1; coactivator associated arginine methyltransferase 1 histone-arginine methyltransferase CARM1-like PRMT6; protein arginine methyltransferase 6

PRMT8; protein arginine methyltransferase 8

PRMT5; protein arginine methyltransferase 5

PRMT9; protein arginine methyltransferase 9

PRMT7; protein arginine methyltransferase 7

**Histone demethylases**

KDM1A; lysine demethylase 1A

KDM1B; lysine demethylase 1B

KDM2B; lysine demethylase 2B

KDM2A; lysine demethylase 2A

KDM3A (JMJD1A); lysine demethylase 3A

KDM3B; lysine demethylase 3B

KDM4A; lysine demethylase 4A

KDM4C; lysine demethylase 4C

JMJD2D (LOC617971); lysine-specific demethylase 4D KDM4B; lysine demethylase 4B

JMJD2D (LOC524768); lysine-specific demethylase 4D

KDM5C; lysine demethylase 5C

KDM5A; lysine demethylase 5A

KDM5B; lysine demethylase 5B

KDM6A; lysine demethylase 6A

UTY; lysine-specific demethylase 6A

KDM6B; lysine demethylase 6B

KDM7A; lysine demethylase 7A

KDM8; lysine demethylase 8

JMJD1C; jumonji domain containing 1C

JMJD6; arginine demethylase and lysine hydroxylase JMJD7; jumonji domain containing 7

JARID2; jumonji and AT-rich interaction domain containing 2

PHF2; PHD finger protein 2

PHF8; PHD finger protein 8

HR; HR, lysine demethylase and nuclear receptor corepressor

RIOX1 (C10H14orf169); ribosomal oxygenase 1

RIOX2 (MINA); ribosomal oxygenase 2

**Histone acetyltransferases**

HAT1; histone acetyltransferase 1

KAT2A; lysine acetyltransferase 2A

KAT2B; lysine acetyltransferase 2B

CREBBP; CREB binding protein

TAF1; TATA-box binding protein associated factor 1

KAT5; lysine acetyltransferase 5

KAT8; lysine acetyltransferase 8

KAT7; lysine acetyltransferase 7

KAT6A; lysine acetyltransferase 6A

KAT6B; lysine acetyltransferase 6B

ELP3; elongator acetyltransferase complex subunit 3

GTF3C4; general transcription factor IIIC subunit 4

NCOA1; nuclear receptor coactivator 1

NCOA2; nuclear receptor coactivator 2

NCOA3; nuclear receptor coactivator 3

CLOCK; clock circadian regulator

CDYL2; chromodomain Y-like 2

CDYL; chromodomain Y-like

NAA40; N(alpha)-acetyltransferase 40, NatD catalytic subunit NAA60; N(alpha)-acetyltransferase 60, NatF catalytic subunit

**Histone deacetylases**

HDAC1; histone deacetylase 1

HDAC2; histone deacetylase 2

HDAC3; histone deacetylase 3

LOC10084903; histone deacetylase 3-like

HDAC8; histone deacetylase 8

HDAC4; histone deacetylase 4

HDAC5; histone deacetylase 5

HDAC6; histone deacetylase 6

HDAC7; histone deacetylase 7

HDAC9; histone deacetylase 9

HDAC10; histone deacetylase 10

HDAC11; histone deacetylase 11

SIRT1; sirtuin 1

SIRT2; sirtuin 2

SIRT3; sirtuin 3

SIRT4; sirtuin 4

SIRT5; sirtuin 5

SIRT6; sirtuin 6

SIRT7; sirtuin 7

**DNA modification proteins**

DNMT1; DNA methyltransferase 1

DNMT3A; DNA methyltransferase 3A

DNMT3B; DNA methyltransferase 3B

DNMT3L; DNA methyltransferase 3L

TET1; Tet methylcytosine Dioxygenase 1

TET2; Tet methylcytosine Dioxygenase 2

TET3; Tet methylcytosine Dioxygenase 3

TDG; Thymine DNA glycosylase
